# Synthesis, crystal structure and Hirshfeld surface analysis of *catena*-poly[[bis­(semicarbazide-κ^2^ *N*,*O*)copper(II)]-μ-sulfato-κ^2^ *O*:*O*′]

**DOI:** 10.1107/S2056989022010040

**Published:** 2022-10-20

**Authors:** Galiya Tadjieva, Aziz B. Ibragimov, Yuldash Yakubov, Bakhtiyar T. Ibragimov, Jamshid M. Ashurov

**Affiliations:** a Institute of General and Inorganic Chemistry of the Uzbekistan Academy of Sciences, M. Ulugbek Str. 77a, Tashkent 700125, Uzbekistan; b Institute of Bioorganic Chemistry Academy of Sciences of Uzbekistan, M. Ulugbek Str. 83, Tashkent 700125, Uzbekistan; Vienna University of Technology, Austria

**Keywords:** semicarbazide, copper complex, crystal structure, Hirshfeld surface analysis, inter­molecular inter­actions

## Abstract

The title polymer, [Cu(SO_4_)(CH_5_N_3_O)_2_]_*n*_, has been synthesized from aqueous solutions of CuSO_4_ and semicarbazide. In the crystal structure, the Cu^II^ atoms are chelated by two neutral semicarbazide mol­ecules through the oxygen atom and a nitro­gen atom of the amino group. The remaining two positions of the Jahn–Teller-distorted octa­hedral coordination sphere are occupied by oxygen atoms of two sulfate anions in the axial positions. The coordination bonds of the latter associate the polyhedra into polymeric chains running parallel to the *c* axis. There is a weak intra­molecular hydrogen bond between the N—H group and an oxygen atom of the SO_4_^2–^ anion. Thirteen relatively weak inter­molecular hydrogen-bonding inter­actions link the chains into a three-dimensional network. Hirshfeld surface analysis revealed that 64.7% of the inter­molecular inter­actions are from O⋯H/H⋯O contacts and 20.1% from H⋯H contacts. Other inter­actions such as N⋯H/H⋯N or C⋯H/H⋯C contribute less to the crystal packing.

## Abstract 2

Coordination bonds of the SO_4_^2–^ anions associate the copper(II) cations into polymeric chains running parallel to the *c* axis.

## 1. Chemical context

Semicarbazide (SEC), a water-soluble white solid, is a deriv­ative of urea with formula O=C(NH_2_)(N_2_H_3_). It is used in the preparation of pharmaceuticals including nitro­furan anti­bacterials (furazolidone, nitro­furazone, nitro­furan­toin) and related compounds (Vass *et al.*, 2008 ▸). Originally, SEC was primarily detected as a nitro­furazone veterinary metabolite, but over time it was found that azodicarbonamide and flour stored in sealed cans could lead to the formation of SEC as well (Tian *et al.*, 2014 ▸). Therefore, the toxicity of SEC as a food contaminant is of crucial inter­est. SEC hydro­chloride has an oral LD_50_ of 225 mg kg^−1^ in mice and 123 mg kg^−1^ in the rat. Some studies suggest that SEC hydro­chloride is a mutagen, an animal carcinogen and a teratogen. As a result of the lack of data in humans and an overall limited evidence of carcinogenicity in animals, SEC was classified by the Inter­national Agency for Research on Cancer as a Group 3 agent, *i.e.* not classifiable as to its carcinogenicity to humans (Takahashi *et al.*, 2014 ▸). However, SEC products (semicarbazones and thiosemicarbazones) are known to have anti­viral, anti-infective and anti­neoplastic activities through binding to copper or iron in cells (Becalski *et al.*, 2004 ▸; Tian *et al.*, 2014 ▸). It is well known that the biopharmaceutical properties of active pharmaceutical ingredients may be improved by metal complex formation (Khudoyberganov *et al.*, 2022 ▸; Ruzmetov *et al.*, 2022*a* ▸,*b* ▸). In turn, this phenomenon may lead to a reduction in the toxicity of haza­rdous organic substances in coordination compounds (Egorova & Ananikov, 2017 ▸; Flora & Pachauri *et al.*, 2010 ▸; Ahmed *et al.*, 2020 ▸). Therefore, it is of great inter­est to study the metal complex formation of SEC. In this context, we report here the synthesis, crystal structure and Hirshfeld surface analysis of a new copper complex of SEC with sulfate anions as co-ligands, [Cu(CH_5_N_3_O)_2_(SO_4_)]_*n*_. 
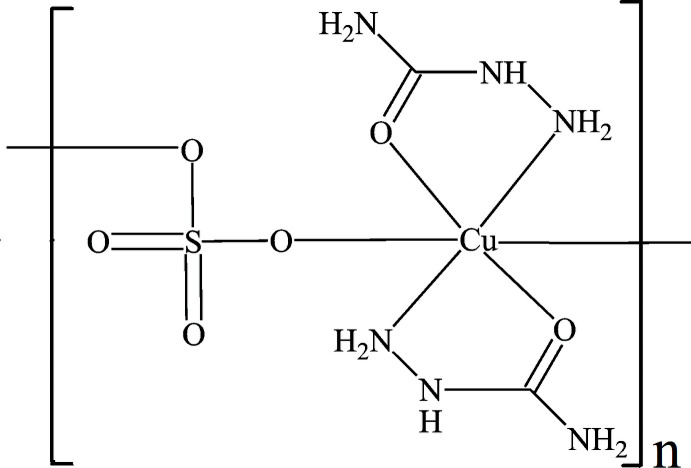


## 2. Structural commentary

**Figure 1:**
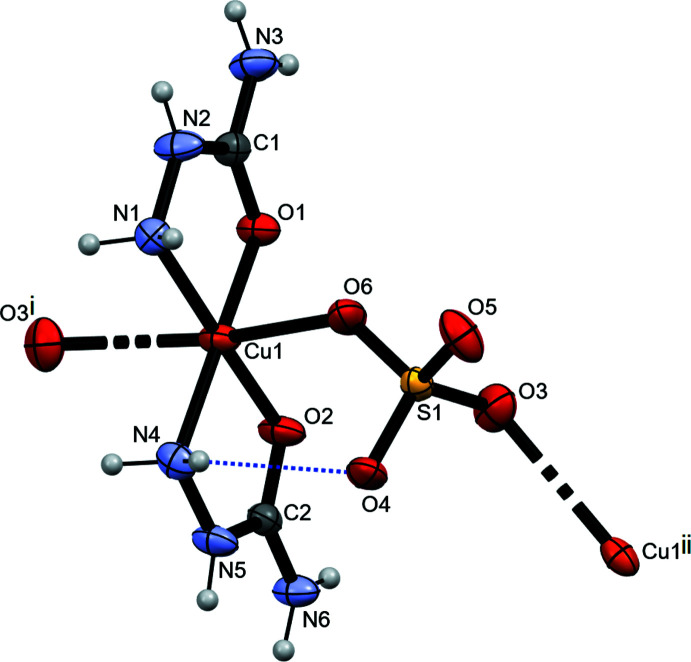
The expanded asymmetric part of the title compound [Cu(SEC)_2_(SO_4_)]_*n*_ with the atom-numbering scheme. The intra­molecular hydrogen bond is indicated by a dashed line. Displacement ellipsoids are plotted at the 50% probability level. [Symmetry codes: (i) *x*, −*y* + 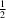 , *z -* 1/2; (ii) *x*, −*y* + 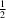 , *z* + 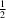 ].

The expanded asymmetric unit of the title polymer is shown in Fig. 1 ▸. The Cu^II^ atom is chelated by two SEC mol­ecules through the oxygen atoms (O1 and O2) and the nitro­gen atoms (N1 and N4) of NH_2_ groups, leading to a slightly distorted square-planar coordination environment with bond lengths in the range between 1.9218 (17) and 2.015 (2) Å and bond angles between 81.50 (7) and 101.89 (8)°. Two remote oxygen atoms, O6 and O3^i^, of two SO_4_^2–^ anions augment the coordination sphere (Table 1 ▸). As a result of the Jahn–Teller effect, a substantial elongation of the two axial Cu—O bonds is observed and the coordination sphere around Cu^II^ becomes a distinctly distorted octa­hedron.

**Figure 2:**
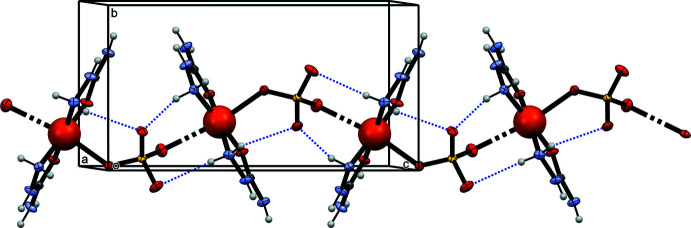
The formation of polymeric chains. Intra­molecular hydrogen bonds are indicated by dotted lines.

Coordination bonds involving the SO_4_^2–^ ligands associate individual polyhedra into polymeric chains running parallel to the *c* axis (Fig. 2 ▸). A weak intra­molecular hydrogen bond between N4—H4 and oxygen atom O4 of the SO_4_ anion (Table 2 ▸), enclosing a six-membered ring with graph-set notation 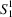 (6) (Etter, 1990 ▸), consolidates the conformation (Fig. 1 ▸). The lengths of the S—O bonds are very similar, showing a distribution between 1.4702 (17) and 1.4769 (17) Å, in very good agreement with the mean value of 1.473 Å for S—O bonds (Gagné & Hawthorne, 2018 ▸).

## 3. Supra­molecular features

**Figure 3:**
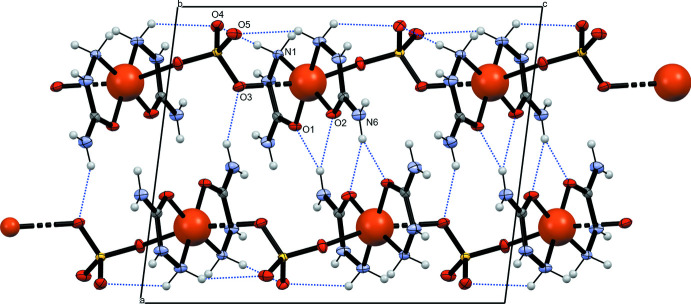
The crystal structure of the title compound. Inter­molecular hydrogen bonds are indicated by dashed lines.

For hydrogen-bonding inter­actions, there are six proton acceptor and ten proton donor functionalities, forming a complex system of 13 inter­molecular hydrogen bonds (Table 2 ▸). Within this network, bifurcated hydrogen bonds involving hydrogen atoms H4*A*, H5 and H6*A* are noted. Each of the atoms O4 and O5 is an acceptor for four hydrogen bonds whereas atoms O1 and O6 are hydrogen-bonded to two hydrogen atoms, and O2 and O3 to one hydrogen atom each. The hydrogen bonds form numerous different associates with various dimensions, *e.g.* there are many rings with graph-set notations ranging from 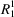 (*n*) to 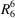 (*n*). The hydrogen bonds indicated in Table 2 ▸ link the polymeric chains into a three-dimensional network (Fig. 3 ▸).

## 4. Hirshfeld surface analysis

**Figure 4:**
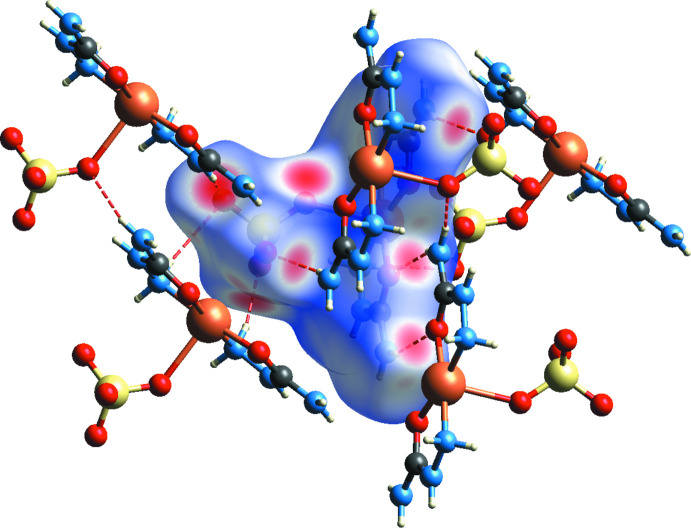
View of the Hirshfeld surface plotted over *d*_norm_.

**Figure 5:**
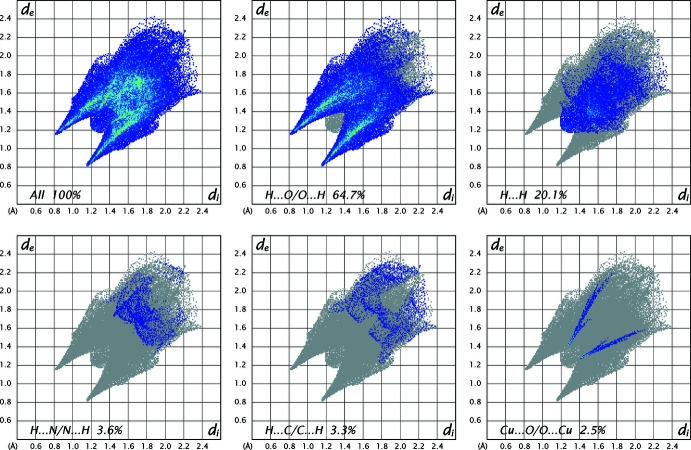
The full two-dimensional fingerprint plot of [Cu(SEC)_2_(SO_4_)]_*n*_ showing all inter­actions and those delineated into O⋯H/H⋯O, H⋯H, N⋯H/H⋯N, C⋯H/H⋯C and C⋯O/O⋯C inter­actions. The *d*_i_ and *d*_e_ values are the closest inter­nal and external distances (Å) from given points on the Hirshfeld surface.

Hirshfeld surfaces were calculated and two-dimensional fingerprints generated using *CrystalExplorer2021* (Spackman *et al.*, 2021 ▸). Fig. 4 ▸ shows the Hirshfeld surface of the title compound with *d*_norm_ (normalized contact distance) plotted over the range −0.5974 to 1.0842 a.u. The inter­actions given in Table 2 ▸ play a key role in the mol­ecular packing of the complex, and nearly two thirds (or 64.7%) of inter­molecular inter­actions correspond to O⋯H/H⋯O contacts The overall two-dimensional fingerprint plot and those delineated into O⋯H/H⋯O, H⋯H, N⋯H/H⋯N, C⋯H/H⋯C and Cu⋯O/O⋯C inter­actions are shown in Fig. 5 ▸. The 2.5% contribution of the Cu⋯O/O⋯Cu contact is explained by the existence of the very long Cu—O3 bond, which is considered by *CrystalExplorer* to be an inter­molecular contact.

## 5. Database survey

A search of the Cambridge Structural Database (CSD, Version 5.43, update of November 2021; Groom *et al.*, 2016 ▸) for semicarbazide metal complexes gave 45 hits. In all entries, neutral semicarbazide mol­ecules coordinate in a chelating fashion enclosing five-membered rings with exception of the Pd complex NAZYES (Bergs *et al.*, 1997 ▸) where a single semicarbazide mol­ecule coordinates monodentately through an NH_2_ group. In 21 mixed-ligand complexes, chloride ions serve as co-ligands except in the structure with refcode SEGWAC (Chuklanova *et al.*, 1988 ▸) where all four ligand positions of the Zn^II^ atom are occupied by Cl^−^ ligands and protonated semicarbazide mol­ecules present as non-coord­inating mol­ecules. Chloride anions likewise are non-coord­inating in four cases, and NO_3_^−^ anions in five structures. Water mol­ecules of crystallization are encountered in 13 complexes. There is only one coordination polymer among the identified compounds, SCACCU10 (Chiesi Villa *et al.*, 1971 ▸). The coordination polyhedron of most of the metal complexes is an octa­hedron while a tetra­hedron is revealed in six cases and penta-coord­ination is found in three structures. Inclusion of the SO_4_^2−^ anion into the coordination sphere of the central metal cation is reported only for the title compound.

## 6. Synthesis and crystallization

0.02 g (0.2 mol) of semicarbazide hydro­chloride, 0.022 g (0.09 mol) of copper sulfate and 0.0054 g (0.09 mol) of mono­ethano­lamine were dissolved separately in 1 ml of water at room temperature. The three solutions were mixed and left in a thermostat at 298 K. After two days, blue crystals started to precipitate. The crystals were filtered off, washed with ethanol and dried.

## 7. Refinement

Crystal data, data collection and structure refinement details are summarized in Table 3 ▸. N-bound hydrogen atoms were placed in calculated positions and refined in the riding-model approximation with *U*_iso_(H) = 1.2*U*_eq_(N), N—H = 0.89 Å for the N1 and N4 nitro­gen atoms and N—H = 0.86 for the remaining nitro­gen atoms.

## Supplementary Material

Crystal structure: contains datablock(s) I. DOI: 10.1107/S2056989022010040/wm5662sup1.cif

Structure factors: contains datablock(s) I. DOI: 10.1107/S2056989022010040/wm5662Isup3.hkl

CCDC reference: 2213165

Additional supporting information:  crystallographic information; 3D view; checkCIF report

## Figures and Tables

**Table 1:** Selected bond lengths (Å)

Cu1—O1	1.9549 (17)	Cu1—N4	2.015 (2)
Cu1—O2	1.9218 (17)	Cu1—O6	2.3776 (18)
Cu1—N1	1.9769 (19)	Cu1—O3^i^	2.6947 (19)

Symmetry code: (i) 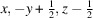 .

**Table 2:** Hydrogen-bond geometry (Å, °)

*D*—H⋯*A*	*D*—H	H⋯*A*	*D*⋯*A*	*D*—H⋯*A*
N1—H1*A*⋯O4^i^	0.89	2.06	2.928 (3)	164
N1—H1*B*⋯O4^ii^	0.89	2.43	3.302 (3)	167
N1—H1*B*⋯O5^ii^	0.89	2.24	2.871 (3)	128
N2—H2⋯O5^iii^	0.86	1.97	2.751 (3)	151
N3—H3*A*⋯O3^iv^	0.86	2.20	2.985 (3)	152
N3—H3*B*⋯O1^v^	0.86	2.59	3.036 (3)	113
N4—H4*A*⋯O4	0.89	2.47	3.125 (3)	131
N4—H4*A*⋯O5^ii^	0.89	2.57	3.097 (3)	119
N4—H4*B*⋯O5^i^	0.89	2.49	3.309 (3)	152
N5—H5⋯O6^vi^	0.86	2.59	3.228 (3)	132
N5—H5⋯O4^vii^	0.86	2.39	2.954 (3)	123
N6—H6*A*⋯O1^viii^	0.86	2.51	3.123 (3)	129
N6—H6*A*⋯O2^viii^	0.86	2.17	2.971 (3)	154
N6—H6*A*⋯O6^vi^	0.86	2.03	2.845 (3)	157

Symmetry codes: (i) 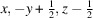 ; (ii) 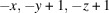 ; (iii) 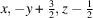 ; (iv) 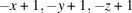 ; (v) 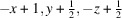 ; (vi) 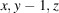 ; (vii) 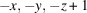 ; (viii) 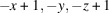 .

**Table 3:** Experimental details

Crystal data	
Chemical formula	[Cu(SO_4_)(CH_5_N_3_O)_2_]
*M*_r_	309.76
Crystal system, space group	Monoclinic, *P*2_1_/*c*
Temperature (K)	293
*a*, *b*, *c* (Å)	10.5555 (2), 6.8624 (1), 12.9061 (2)
β (°)	97.265 (2)
*V* (Å^3^)	927.36 (3)
*Z*	4
Radiation type	Cu *K*α
μ (mm^−1^)	5.82
Crystal size (mm)	0.18 × 0.16 × 0.14

Data collection	
Diffractometer	XtaLAB Synergy, Single source at home/near, HyPix3000
Absorption correction	Multi-scan (*CrysAlis PRO*; Rigaku, 2020 ▸)
*T*_min_, *T*_max_	0.084, 1.000
No. of measured, independent and observed [*I* > 2σ(*I*)] reflections	8102, 1785, 1656
*R*_int_	0.037
(sin θ/λ)_max_ (Å^−1^)	0.613

Refinement	
*R*[*F* ^2^ > 2σ(*F* ^2^)], *wR*(*F* ^2^), *S*	0.029, 0.080, 1.07
No. of reflections	1785
No. of parameters	146
H-atom treatment	H-atom parameters constrained
Δρ_max_, Δρ_min_ (e Å^−3^)	0.32, −0.42

Computer programs: *CrysAlis PRO* (Rigaku, 2020 ▸), SHELXT (Sheldrick, 2015*a* ▸), *SHELXL* (Sheldrick, 2015*b* ▸) and *OLEX2* (Dolomanov *et al.*, 2009 ▸).

## supplementary crystallographic information

### Crystal data

**Table:**

[Cu(SO_4_)(CH_5_N_3_O)_2_]	*F*(000) = 628
*M**_r_* = 309.76	*D*_x_ = 2.219 Mg m^−^^3^
Monoclinic, *P*2_1_/*c*	Cu *K*α radiation, λ = 1.54184 Å
*a* = 10.5555 (2) Å	Cell parameters from 4840 reflections
*b* = 6.8624 (1) Å	θ = 3.5–70.8°
*c* = 12.9061 (2) Å	µ = 5.82 mm^−^^1^
β = 97.265 (2)°	*T* = 293 K
*V* = 927.36 (3) Å^3^	Block, light blue
*Z* = 4	0.18 × 0.16 × 0.14 mm

### Data collection

**Table:**

XtaLAB Synergy, Single source at home/near, HyPix3000 diffractometer	1785 independent reflections
Radiation source: micro-focus sealed X-ray tube, PhotonJet (Cu) X-ray Source	1656 reflections with *I* > 2σ(*I*)
Mirror monochromator	*R*_int_ = 0.037
Detector resolution: 10.0000 pixels mm^-1^	θ_max_ = 71.1°, θ_min_ = 4.2°
ω scans	*h* = −12→12
Absorption correction: multi-scan (CrysAlisPro; Rigaku, 2020)	*k* = −8→6
*T*_min_ = 0.084, *T*_max_ = 1.000	*l* = −15→15
8102 measured reflections	

### Refinement

**Table:**

Refinement on *F*^2^	Hydrogen site location: inferred from neighbouring sites
Least-squares matrix: full	H-atom parameters constrained
*R*[*F*^2^ > 2σ(*F*^2^)] = 0.029	*w* = 1/[σ^2^(*F*_o_^2^) + (0.0437*P*)^2^ + 0.5652*P*] where *P* = (*F*_o_^2^ + 2*F*_c_^2^)/3
*wR*(*F*^2^) = 0.080	(Δ/σ)_max_ < 0.001
*S* = 1.07	Δρ_max_ = 0.32 e Å^−^^3^
1785 reflections	Δρ_min_ = −0.41 e Å^−^^3^
146 parameters	Extinction correction: SHELXL-2018/3 (Sheldrick, 2015b), Fc^*^=kFc[1+0.001xFc^2^λ^3^/sin(2θ)]^-1/4^
0 restraints	Extinction coefficient: 0.0019 (3)

### Special details

**Table:**

Geometry. All esds (except the esd in the dihedral angle between two l.s. planes) are estimated using the full covariance matrix. The cell esds are taken into account individually in the estimation of esds in distances, angles and torsion angles; correlations between esds in cell parameters are only used when they are defined by crystal symmetry. An approximate (isotropic) treatment of cell esds is used for estimating esds involving l.s. planes.

### Fractional atomic coordinates and isotropic or equivalent isotropic displacement parameters (Å^2^)

**Table:**

	*x*	*y*	*z*	*U*_iso_*/*U*_eq_	
Cu1	0.25619 (3)	0.28648 (5)	0.38274 (3)	0.02624 (15)	
S1	0.15524 (5)	0.43123 (8)	0.62353 (4)	0.02198 (17)	
O1	0.40378 (15)	0.4485 (2)	0.36355 (13)	0.0265 (4)	
O2	0.36126 (16)	0.0997 (2)	0.46464 (15)	0.0320 (4)	
O4	0.06548 (18)	0.2652 (3)	0.61509 (14)	0.0327 (4)	
O3	0.26979 (17)	0.3862 (3)	0.69698 (15)	0.0390 (5)	
N1	0.16194 (18)	0.4839 (3)	0.29178 (15)	0.0240 (4)	
H1A	0.129486	0.430444	0.231376	0.029*	
H1B	0.098181	0.533034	0.322557	0.029*	
N4	0.11789 (19)	0.0915 (3)	0.40077 (17)	0.0284 (4)	
H4A	0.057785	0.146007	0.433787	0.034*	
H4B	0.081860	0.049058	0.338815	0.034*	
N5	0.1763 (2)	−0.0647 (3)	0.46025 (18)	0.0311 (5)	
H5	0.132873	−0.161448	0.479156	0.037*	
N2	0.2496 (2)	0.6315 (3)	0.27463 (18)	0.0333 (5)	
H2	0.225589	0.736581	0.241505	0.040*	
N6	0.3650 (2)	−0.2015 (3)	0.53428 (18)	0.0318 (5)	
H6A	0.446089	−0.194830	0.552441	0.038*	
H6B	0.323773	−0.304631	0.547814	0.038*	
N3	0.4573 (2)	0.7341 (3)	0.29420 (19)	0.0369 (5)	
H3A	0.536565	0.718013	0.317642	0.044*	
H3B	0.433312	0.836957	0.258968	0.044*	
C1	0.3723 (2)	0.6007 (3)	0.31234 (18)	0.0238 (5)	
C2	0.3037 (2)	−0.0535 (3)	0.48563 (18)	0.0240 (5)	
O5	0.09208 (18)	0.6011 (3)	0.66402 (17)	0.0407 (5)	
O6	0.19228 (18)	0.4786 (3)	0.52040 (13)	0.0333 (4)	

### Atomic displacement parameters (Å^2^)

**Table:**

	*U*^11^	*U*^22^	*U*^33^	*U*^12^	*U*^13^	*U*^23^
Cu1	0.0189 (2)	0.0213 (2)	0.0375 (2)	−0.00162 (12)	−0.00018 (15)	0.00774 (14)
S1	0.0190 (3)	0.0238 (3)	0.0234 (3)	−0.0045 (2)	0.0034 (2)	−0.0019 (2)
O1	0.0184 (8)	0.0231 (8)	0.0378 (9)	0.0007 (6)	0.0025 (7)	0.0065 (7)
O2	0.0225 (9)	0.0225 (8)	0.0491 (11)	−0.0053 (7)	−0.0035 (8)	0.0109 (8)
O4	0.0322 (10)	0.0292 (9)	0.0356 (10)	−0.0134 (7)	0.0001 (8)	0.0053 (7)
O3	0.0233 (9)	0.0524 (12)	0.0391 (10)	−0.0035 (8)	−0.0043 (8)	0.0060 (9)
N1	0.0187 (10)	0.0263 (10)	0.0271 (10)	0.0015 (8)	0.0036 (8)	−0.0003 (8)
N4	0.0196 (10)	0.0276 (10)	0.0374 (11)	−0.0021 (8)	0.0007 (8)	−0.0011 (9)
N5	0.0251 (11)	0.0225 (9)	0.0453 (12)	−0.0067 (8)	0.0030 (9)	0.0045 (9)
N2	0.0246 (11)	0.0277 (11)	0.0468 (13)	0.0013 (8)	0.0014 (9)	0.0164 (10)
N6	0.0282 (12)	0.0234 (10)	0.0432 (13)	−0.0014 (8)	0.0016 (9)	0.0102 (9)
N3	0.0319 (13)	0.0342 (12)	0.0446 (13)	−0.0088 (9)	0.0050 (10)	0.0145 (10)
C1	0.0240 (12)	0.0231 (11)	0.0256 (11)	0.0011 (9)	0.0086 (9)	0.0000 (9)
C2	0.0250 (12)	0.0212 (11)	0.0262 (11)	−0.0032 (9)	0.0040 (9)	−0.0011 (9)
O5	0.0284 (10)	0.0392 (10)	0.0548 (12)	−0.0009 (8)	0.0065 (9)	−0.0220 (9)
O6	0.0425 (11)	0.0316 (9)	0.0276 (9)	−0.0112 (8)	0.0115 (8)	−0.0017 (7)

### Geometric parameters (Å, º)

**Table:**

Cu1—O1	1.9549 (17)	N1—N2	1.408 (3)
Cu1—O2	1.9218 (17)	N4—H4A	0.8900
Cu1—N1	1.9769 (19)	N4—H4B	0.8900
Cu1—N4	2.015 (2)	N4—N5	1.414 (3)
Cu1—O6	2.3776 (18)	N5—H5	0.8600
Cu1—O3^i^	2.6947 (19)	N5—C2	1.345 (3)
S1—O4	1.4769 (17)	N2—H2	0.8600
S1—O3	1.4719 (18)	N2—C1	1.341 (3)
S1—O5	1.4714 (19)	N6—H6A	0.8600
S1—O6	1.4702 (17)	N6—H6B	0.8600
O1—C1	1.258 (3)	N6—C2	1.320 (3)
O2—C2	1.261 (3)	N3—H3A	0.8600
N1—H1A	0.8900	N3—H3B	0.8600
N1—H1B	0.8900	N3—C1	1.324 (3)
			
O1—Cu1—N1	83.36 (7)	N2—N1—H1A	110.3
O1—Cu1—N4	173.06 (8)	N2—N1—H1B	110.3
O1—Cu1—O6	94.90 (7)	Cu1—N4—H4A	110.3
O2—Cu1—O1	92.02 (7)	Cu1—N4—H4B	110.3
O2—Cu1—N1	174.70 (8)	H4A—N4—H4B	108.6
O2—Cu1—N4	82.52 (8)	N5—N4—Cu1	107.04 (14)
O2—Cu1—O6	99.05 (7)	N5—N4—H4A	110.3
N1—Cu1—N4	101.89 (8)	N5—N4—H4B	110.3
N1—Cu1—O6	83.95 (7)	N4—N5—H5	121.8
N4—Cu1—O6	90.21 (8)	C2—N5—N4	116.32 (19)
O2—Cu1—O3^i^	96.00 (7)	C2—N5—H5	121.8
O1—Cu1—O3^i^	90.24 (7)	N1—N2—H2	121.5
N1—Cu1—O3^i^	81.50 (7)	C1—N2—N1	116.94 (19)
N4—Cu1—O3^i^	86.13 (9)	C1—N2—H2	121.5
O6—Cu1—O3^i^	163.90 (6)	H6A—N6—H6B	120.0
O3—S1—O4	110.64 (11)	C2—N6—H6A	120.0
O5—S1—O4	108.79 (11)	C2—N6—H6B	120.0
O5—S1—O3	108.05 (12)	H3A—N3—H3B	120.0
O6—S1—O4	110.20 (10)	C1—N3—H3A	120.0
O6—S1—O3	109.80 (11)	C1—N3—H3B	120.0
O6—S1—O5	109.31 (12)	O1—C1—N2	120.0 (2)
C1—O1—Cu1	112.13 (15)	O1—C1—N3	121.8 (2)
C2—O2—Cu1	114.46 (15)	N3—C1—N2	118.2 (2)
Cu1—N1—H1A	110.3	O2—C2—N5	119.3 (2)
Cu1—N1—H1B	110.3	O2—C2—N6	121.6 (2)
H1A—N1—H1B	108.5	N6—C2—N5	119.1 (2)
N2—N1—Cu1	107.15 (14)	S1—O6—Cu1	133.39 (11)
			
Cu1—O1—C1—N2	−2.6 (3)	O3—S1—O6—Cu1	76.86 (17)
Cu1—O1—C1—N3	177.26 (19)	N1—N2—C1—O1	−2.4 (3)
Cu1—O2—C2—N5	−7.0 (3)	N1—N2—C1—N3	177.7 (2)
Cu1—O2—C2—N6	174.70 (19)	N4—N5—C2—O2	6.7 (3)
Cu1—N1—N2—C1	5.9 (3)	N4—N5—C2—N6	−175.0 (2)
Cu1—N4—N5—C2	−2.9 (2)	O5—S1—O6—Cu1	−164.78 (14)
O4—S1—O6—Cu1	−45.26 (18)		

Symmetry code: (i) *x*, −*y*+1/2, *z*−1/2.

### Hydrogen-bond geometry (Å, º)

**Table:**

*D*—H···*A*	*D*—H	H···*A*	*D*···*A*	*D*—H···*A*
N1—H1*A*···O4^i^	0.89	2.06	2.928 (3)	164
N1—H1*B*···O4^ii^	0.89	2.43	3.302 (3)	167
N1—H1*B*···O5^ii^	0.89	2.24	2.871 (3)	128
N2—H2···O5^iii^	0.86	1.97	2.751 (3)	151
N3—H3*A*···O3^iv^	0.86	2.20	2.985 (3)	152
N3—H3*B*···O1^v^	0.86	2.59	3.036 (3)	113
N4—H4*A*···O4	0.89	2.47	3.125 (3)	131
N4—H4*A*···O5^ii^	0.89	2.57	3.097 (3)	119
N4—H4*B*···O5^i^	0.89	2.49	3.309 (3)	152
N5—H5···O6^vi^	0.86	2.59	3.228 (3)	132
N5—H5···O4^vii^	0.86	2.39	2.954 (3)	123
N6—H6*A*···O1^viii^	0.86	2.51	3.123 (3)	129
N6—H6*A*···O2^viii^	0.86	2.17	2.971 (3)	154
N6—H6*A*···O6^vi^	0.86	2.03	2.845 (3)	157

Symmetry codes: (i) *x*, −*y*+1/2, *z*−1/2; (ii) −*x*, −*y*+1, −*z*+1; (iii) *x*, −*y*+3/2, *z*−1/2; (iv) −*x*+1, −*y*+1, −*z*+1; (v) −*x*+1, *y*+1/2, −*z*+1/2; (vi) *x*, *y*−1, *z*; (vii) −*x*, −*y*, −*z*+1; (viii) −*x*+1, −*y*, −*z*+1.
